# Intravenous fluid therapy: a multi-national, cross-sectional survey of common medical student resources

**DOI:** 10.1186/s12909-022-03433-4

**Published:** 2022-06-14

**Authors:** Jack B. Ding, Thomas C. Varkey

**Affiliations:** 1 Adelaide Medical School, University of Adelaide, SA Adelaide, Australia; 2 Grand Canyon University, Phoenix, Arizona USA; 3 College of Medicine, The University of Arizona, Phoenix, Arizona USA

**Keywords:** Intravenous fluid therapy, Medical education, Medical student resources

## Abstract

**Background:**

Inappropriate prescription of intravenous fluid therapy is highly prevalent in hospitals, with up to 1 in 5 patients suffering from preventable, additional morbidity. Since trainee physicians are frequently responsible for prescribing intravenous fluids, it is possible that common medical student resources do not sufficiently cover the topic. There is a paucity of recent literature on this issue, which this study was designed to address.

**Methods:**

Two original evaluation tools were created by the authors to evaluate reference books, official guidelines, and online reference sources commonly used by medical students in the United States of America, Australia, and the United Kingdom on their coverage of foundational and clinically relevant principles of intravenous fluid prescription. The choice of student resources was guided by a literature search and personal experience. A total of 10 resources was assessed.

**Results:**

Resources were generally deficit in their coverage of basic intravenous fluid topics. The total points each topic accumulated ranged from 0.5 (5%) to 7.5 (75%), with the median score being 4.5 (45%), on a scale from 0 to 10 points.

**Conclusions:**

Popular medical student resources poorly cover intravenous fluid therapy topics. This may be contributing to inadequate fluid prescribing practices.

**Supplementary Information:**

The online version contains supplementary material available at 10.1186/s12909-022-03433-4.

## Introduction

Fluid infusion therapy is ubiquitous in the inpatient setting, with over 200 million liters of saline prescribed annually in the United States of America (US) alone [1]. Suboptimal prescription of intravenous (IV) fluid therapy is commonly encountered, with the 2013 National Institute for Health and Care Excellence (NICE) suggesting that up to 1 in 5 patients likely suffer from additional morbidity as a consequence of inappropriate fluid prescription practices [2]. A prospective study of 71 patients who were prescribed IV fluids discovered that 7 patients experienced a tachyarrhythmia, and 5 patients were clinically fluid overloaded by the end of fluid therapy [3].

The responsibility of prescribing fluid therapy often rests with trainee physicians, such as interns and residents. This is despite the wide disparities in competency displayed by junior medical staff with regards to appropriate IV fluid prescribing and documentation practices [4]. A 2018 British survey of 143 junior medical doctors discovered that only 45% were able to correctly recall an otherwise healthy adult’s daily maintenance fluid requirement (25 to 30 mls/kg/day), and that only 37% correctly stated the concentration of sodium in 0.9% NaCl [5].

One could argue that intravenous fluid prescribing is a practical skill to be learnt ‘on the job’. However, clinicians and researchers are increasingly describing fluids as ‘drugs’, with their own set of quantifiable efficacies and toxicities [6]. Moreover, remedial interventions are frequently implemented in teaching hospitals for junior residents to compensate for knowledge deficiencies, with the bulk of content relating to foundational knowledge regarding fluid and electrolytes, rather than practical knowledge relating to fluid prescription [7]. This could suggest that the primary knowledge deficit is due to ameliorable breaches in preliminary medical education in the clinical years of medical school rather than systematic inefficiencies that improve with familiarity.

Another issue with trainee physicians learning IV fluid therapy prescribing on the job is that no medical intervention is without conveying a certain level of risk to the patient. Medical schools often reach significant depth with their basic science education of commonly prescribed drugs, and the interactions these drugs may have on overarching physiologic systems. IV fluids are deceptively complex, and can influence electrolyte balances, haemodynamic parameters, acid–base balance, and all organ systems. The difference between optimal IV fluid therapy and devastating outcome may hinge on a single dynamic, such as rate of infusion in the case of acute pulmonary edema or pontine osmotic demyelinating syndrome. [8, 9] Yet, IV fluid therapy education is often not covered in comparable detail to other less commonly prescribed drugs.

This study aims to assess whether current medical student learning resources are insufficient in their coverage of foundational IV fluid knowledge. If so, this could at least partially account for the knowledge deficiencies some junior residents have with IV fluid prescribing practice. Recognizing the centrality of context in medical education, we synchronize our evaluative approach with the increasing normalization and pervasiveness of self-directed learning by assessing the medical school learning resources most used by clinical medical students from the US, United Kingdom, and Australia, as reported in the literature and through the personal experiences of the authors. This is instead of evaluating authoritative medical textbooks or resources recommended by medical school for learning, which may not be appropriately representative of actual resource use patterns [10].

## Materials and methods

Prior to commencing the survey, the authors created two original evaluation tools for assessing student resources. An extensive list of potential topics was first created by the authors following a review of the literature on the topic, which were then selected for inclusion based on their clinical relevance for a medical intern. Relevance was determined through consultation with senior physicians, interns, medical students, and the clinical experience of the authors, who both have a special interest on the subject. Both tools are checklists consisting of common concepts related to fluid prescription practices, with Tool 1 covering basic knowledge and Tool 2 covering clinical knowledge. Resources were scored based on whether they covered everything in the checklist (1 point), some of it (0.5 points) or none of it (0 points). The maximum possible points each topic on Tool 1 and Tool 2 could score was 10 points.

### List of topics included in tool 1

(1): Fluid balance: normal input; normal output; (2): Fluid balance: causes of altered output in a clinical setting; (3): Fluid status physical findings: hypervolemia, hypovolemia; (4): Electrolyte maintenance input for: Na, Cl, K; (5): Cannulas: basic indications for gaining central vs peripheral access; (6): 0.9% NaCl: electrolyte content, tonicity, osmolarity; (7): Ringer’s lactate: electrolyte content, tonicity, osmolarity; (8): 0.45% NaCl: electrolyte content, tonicity, osmolarity; (9): D5W: electrolyte content, tonicity, osmolarity; (10): Albumin: content, tonicity, osmolarity; (11): Semi-synthetic colloids: content, tonicity, osmolarity; (12): Risks of hypertonic IV fluids (mild, moderate, critical severity); (13): Risks of hypotonic IV fluids (mild, moderate, critical severity).

### List of topics included in tool 2

(1): ‘Bolus’ IV fluid: indications, choice of fluid, volume, rate, when to stop; (2): Maintenance IV fluid when NPO: indications, choice of fluid, volume, rate, when to stop; (3): Fluid restriction: indications, volume, timing, supplementation, when to stop; (4): Fluid resuscitation in the setting of blood loss: choice of fluid, volume, rate, when to stop; (5): Fluid resuscitation in the setting of GI loss: choice of fluid, volume, rate, when to stop; (6): IV fluid challenge: indications, choice of fluid, volume, rate, when to stop; (7): Use of crystalloids and blood products in the setting of shock.

Following this, the authors conducted a literature search for recent, high-quality evaluations of clinical year medical students and their self-reported primary study resources. Data was extracted from four studies [11–14], which the authors used in conjunction with their personal experiences to select resources that provided comparable and realistic representation of the total resource pool for Australian, American, and British medical students. Table 1 below is an overview of selected student resources.

**Table 1:** Medical student resources selected for analysis in this study

**Reference books**			
Title	Edition	Year	Publisher
First Aid for Step 2CK [15]	Tenth	2018	McGraw-Hill Education
Oxford Handbook of Clinical Medicine [16]	Ninth	2014	Oxford University Press
Toronto Notes [17]	Thirty sixth	2020	Toronto Notes for Medical Students, Inc
Step-Up to Step 2CK [18]	Fourth	2016	Wolters Kluwer
Master the Boards [19]	Fourth	2017	Kaplan Publishing
**Online guidelines**			
**Database**	**Access Date**		**Publisher**
UpToDate	September 2021		Wolters Kluwer Health
NICE Guidelines	October 2021		National Institute for Health and Care Excellence
Therapeutic guidelines (eTG)	October 2021		Therapeutic guidelines limited
**Online resources**			
**Resource**	**Access Date**	**Resource type**	**Publisher**
AMBOSS	2021	MCQ bank and digital library	AMBOSS GmbH
OnlineMedEd	2021	Video lectures	OnlineMedEd

*NICE* National Institute for Health and Care Excellence

The resources were then independently evaluated by one author. To assess the reliability of scoring, a second blinded author independently assessed a resource that was selected through a random generator. The two scorings were then compared, and Spearman’s rank correlation coefficient was subsequently calculated.

## Results

The scoring comparison between the two independent evaluators yielded a Spearman coefficient of Rank concordance of 0.9425 (*p* = 0.001).

The range of distributed scores among all topics was 0.5 (5%) to 7.5 (75%), with the median score being 4.5 (45%). As there were 10 resources, and the possible evaluation scores were 0, 0.5, and 1 point, the total possible points each topic could achieve was 10. Evaluation Tool 1 was the checklist used to evaluate basic knowledge, while Evaluation Tool 2 evaluated clinical knowledge. Finally, Group 3 is the combination of points topics from both tools accumulated. Table 2 displays a summary of collated data. Groups 1 and 2 display the total points each topic from Evaluation Tool 1 and 2 accumulated after all ten resources were evaluated, respectively. Table 3 displays all the topics of Evaluation Tool 1 and 2 and the aggregate points awarded to each topic following evaluation of all ten resources.

**Table 2:** Performance summary of each topic grouping

Group	Min	Q_1_	Median	Q_3_	Max
1	2	4	4.5	6	7.5
2	0.5	4.5	5.5	6	6
3	0.5	4.5	4.5	6	7.5

Group 1: total points each basic knowledge topic from Evaluation Tool 1 received (maximum 10), Group 2: total points each clinical knowledge topic from Evaluation Tool 2 received (maximum 10), Group 3: total points of all topics from both Evaluation Tool 1 and 2

**Table 3:** List of topics in Evaluation Tool 1 and 2 and the total points they each accumulated after all ten resources were evaluated with the tools

**List of topics in Tool 1 (basic knowledge)**	**Points (max 10)**
Fluid balance: normal input; normal output	7.5
Fluid balance: causes of altered output in a clinical setting	7
Fluid status physical findings: hypervolemia, hypovolemia	7.5
Electrolyte maintenance input for: Na, Cl, K	3.5
Cannulas: basic indications for gaining central vs peripheral access	2.5
0.9% NaCl: electrolyte content, tonicity, osmolarity	5
Ringer’s lactate: electrolyte content, tonicity, osmolarity	4.5
0.45% NaCl: electrolyte content, tonicity, osmolarity	4.5
D5W: electrolyte content, tonicity, osmolarity	4.5
Albumin: content, tonicity, osmolarity	2
Semi-synthetic colloids: content, tonicity, osmolarity	4.5
Risks of hypertonic IV fluids	4.5
Risks of hypotonic IV fluids	4.5
**List of topics in Tool 2 (clinical knowledge)**	**Points (max 10)**
‘Bolus’ IV fluid: indications, choice of fluid, volume, rate, when to stop	6
Maintenance IV fluid when NPO: indications, choice of fluid, volume, rate, when to stop	4.5
Fluid resuscitation in the setting of blood loss: choice of fluid, volume, rate, when to stop	6
Fluid resuscitation in the setting of GI loss: choice of fluid, volume, rate, when to stop	6
IV fluid challenge: indications, choice of fluid, volume, rate, when to stop	5.5
Fluid restriction: Volume to restrict, timing, utilization of supplementation, indications, when to stop	5
Crystalloids vs Blood products	0.5

## Discussion

There is comparable representation of practical content and knowledge base content across resources, with the pooled median score of the resources of each country being 4.5 (45%) for knowledge-based topics, and 5.5 (55%) for practical topics. As seen by the percentage scoring, the depth of coverage is globally deficient.

Notably, the highest performing medical student book, Toronto Notes, scored only 12.5/20 (62.5%). This is concerning, as at least one medical student book from each country that is regarded as an unofficial ‘gold standard’ from each country was included in the resources selected for analysis.

Surprisingly, only one of the three guidelines provided comprehensive coverage of topics, with a total score of 19 (95%), whereas the other two guidelines only scored 11.5 (57.5%) and 13 (65%), which is inferior to the commercial AMBOSS online medical student reference resource, which scored 15 (75%) (see Supplementary Tables 1 and 2). This may be because official guidelines are intended for refreshing the more senior clinician with the latest evidence-backed suggestions, and therefore a significant amount of foundational knowledge may be presumed and not mentioned in sufficient detail to warrant the ‘All covered’ outcome in the scoring process. From a medical student resource perspective, official guidelines may not be presented in the most readily consumable manner for learning, which may necessitate simpler perspectives. For instance, first principles on fluid expansion may be obfuscated by up-to-date primary research and discussions of counterintuitive controversies, such as how colloid products may possibly increase mortality in certain fluid depleted patients. Therefore, students may not even use higher performing guidelines for their first walkthrough of the topic. And if they do, the efficiency of their time may be decreased.

Discussion on crystalloids and blood products in the setting of shock was covered the worst, with only 0.5 points, meaning it was only superficially covered by one of the resources out of the pool of 10 possible resources from 3 countries. Albumin and other colloid products were also poorly covered, with the topic scoring only 2 points (22%). This is likely because its widespread use has fallen out of favor due to concerns with increasing mortality rate, though there are still a few specific indications that support its use. A comparison of indications for peripheral and central cannulas also only scored 2 points. This was surprising as this topic was considered fundamental for the intravenous delivery of any product. All the abovementioned topics are clinically relevant and commonly encountered in general medicine and surgical wards.

Since all the non-guideline resources parallel local medical student examination standards, their content provides a reasonable barometer for the depth and likelihood of any given topic’s appearance in live examination format. We therefore deduce from this that the current appearance of IV fluid therapy in exams is likely limited in breadth and depth. Therefore, we call for increasing IV fluid therapy representation for the trainees’ and patients’ benefit. This could trigger a significant change of the topic in non-guideline medical student resources. The newly evolved medical education landscape may then better transition the medical student into their role as a physician in training, as they will be better equipped with the basic knowledge to manage some of the most common prescriptions in hospital. Beyond that, IV fluid therapy related patient complications may potentially decrease.

Our international approach in surveying the IV fluid therapy medical education landscape increases suspicion that neglect of this topic may be globally pervasive rather than isolated to a particular geographic region. Indeed, there is evidence that IV fluid prescription is sub-optimally performed in countries without a mainly English-speaking population, with one Iranian study of 450 inpatients who received IV fluids reporting a prescribing error rate of 1.3% per patient. [20] Further evaluation of medical education resources and curriculums from an international perspective may help determine the extent of this issue, and the potential for medical educators to bridge this deficiency at the medical school level.

### Limitations

The primary limitation of this study is that it did not account for content directly derived from medical school curricula, which is likely to be heterogenous in content and quality [21].

For each country, one top resource was selected from each following categories: reference book, online guidelines, and online reference source. However, the culture of learning may be different between countries, with physical textbooks being preferred by UK students for certain clinical studies, whereas online resources may be more popular for US students [14]. Therefore, while using this data for comparison purposes between countries is likely not useful, the international data set best demonstrates that the deficiency of coverage is not necessarily localized to just one part of the world.

## Conclusions

IV fluids are among the most frequently prescribed products in hospitals. Inappropriate IV fluid prescription practices are common and contribute to substantial, costly, and preventable patient morbidity and mortality. The most frequently used medical student resources in the United States, United Kingdom, and Australia do not adequately cover fundamental topics related to IV fluid prescribing. There is no evidence to suggest that this deficiency is restricted to these three countries. The authors suggest that a possible contributing reason may be due to underemphasis of IV fluid prescribing in examination settings. This in turn can lessen the resolve of resource creators to detail the topic in further detail, as the top resources in all three countries tend to be highly succinct and focused on material most likely to appear in examinations. Further studies on IV fluid prescription practices and medical student resources on the topic in other countries can help determine the degree to which this issue extends to on a global scale.

## Supplementary Information

**Additional file 1.**

## Data Availability

Data is available as a supplementary file.
