# Supplementary material for: Intravenous fluid therapy: a multi-national, cross-sectional survey of common medical student resources
Source: BMC Med Educ. 2022 Jun 14;22:454. doi: 10.1186/s12909-022-03433-4 (PMC9195194; doi:10.1186/s12909-022-03433-4)
Supplement: Supplementary file 1 — Additional file 1. [file 12909_2022_3433_MOESM1_ESM.docx]

Supplementary Table 1. Points each resource received per topic on Evaluation Tool 1

| TOPIC | OME | FA | U2D | OX | NICE | TN | AB | ETG | SU | MTB | TOTAL |
| --- | --- | --- | --- | --- | --- | --- | --- | --- | --- | --- | --- |
| Fluid balance: normal input; normal output | 1 | 1 | 1 | 1 | 1 | 1 | 1 | 0.5 | 0 | 0 | 7.5 |
| Fluid balance: causes of altered output in a clinical setting | 0.5 | 1 | 1 | 0 | 1 | 1 | 1 | 1 | 0 | 0.5 | 7 |
| Fluid status physical findings: hypervolemia, hypovolemia | 1 | 0.5 | 1 | 0 | 1 | 1 | 1 | 1 | 0.5 | 0.5 | 7.5 |
| Electrolyte maintenance input for: Na, Cl, K | 0 | 0 | 1 | 0 | 1 | 1 | 0 | 0.5 | 0 | 0 | 3.5 |
| Cannulas: basic indications for gaining central vs peripheral access | 0.5 | 0 | 1 | 0 | 0 | 0 | 1 | 0 | 0 | 0 | 2.5 |
| 0.9% NaCl: electrolyte content, tonicity, osmolarity | 0 | 0 | 1 | 0.5 | 1 | 1 | 1 | 0.5 | 0 | 0 | 5 |
| Ringer’s lactate: electrolyte content, tonicity, osmolarity | 0 | 0 | 1 | 0 | 1 | 1 | 1 | 0.5 | 0 | 0 | 4.5 |
| 0.45% NaCl: electrolyte content, tonicity, osmolarity | 0 | 0 | 1 | 0 | 1 | 1 | 1 | 0.5 | 0 | 0 | 4.5 |
| D5W: electrolyte content, tonicity, osmolarity | 0 | 0 | 1 | 0.5 | 1 | 1 | 1 | 0 | 0 | 0 | 4.5 |
| Albumin: content, tonicity, osmolarity | 0 | 0 | 1 | 0 | 0 | 0 | 1 | 0 | 0 | 0 | 2 |
| Semi-synthetic colloids: content, tonicity, osmolarity | 0 | 0 | 1 | 0.5 | 1 | 1 | 1 | 0 | 0 | 0 | 4.5 |
| Risks of hypertonic IV fluids | 0.5 | 0.5 | 1 | 0 | 0.5 | 0 | 1 | 0.5 | 0 | 0.5 | 4.5 |
| Risks of hypotonic IV fluids | 0.5 | 0.5 | 1 | 0 | 0.5 | 0 | 1 | 0.5 | 0 | 0.5 | 4.5 |
| TOTAL | 4 | 3.5 | 13 | 2.5 | 10 | 9 | 12 | 5.5 | 0.5 | 2 | 62 |
| Legend: AB: AMBOSS; ETG: Electronic Therapeutic Guidelines; FA: First Aid; MTB: Master the Boards; NICE: National Institute for Health and Care Excellence; OME: OnlineMedEd; OX: Oxford Handbook of Clinical Medicine; SU: Step-Up to Step 2CK; TN: Toronto Notes  There were three possible scorings for each topic: 0 point (none covered), 0.5 points (some covered), 1 point (all covered.  Supplementary Table 2. Points each resource received per topic on Evaluation Tool 2 | | | | | | | | | | |  |
| TOPIC | OME | FA | U2D | OX | NICE | TN | AB | ETG | SU | MTB | Total |
| ‘Bolus’ IV fluid: indications, choice of fluid, volume, rate, when to stop | 0.5 | 0.5 | 1 | 0.5 | 0.5 | 0.5 | 0.5 | 1 | 0.5 | 0.5 | 6 |
| Maintenance IV fluid when NPO: indications, choice of fluid, volume, rate, when to stop | 0.5 | 0 | 1 | 0 | 0.5 | 0.5 | 0.5 | 1 | 0 | 0.5 | 4.5 |
| Fluid resuscitation in the setting of blood loss: choice of fluid, volume, rate, when to stop | 0.5 | 0.5 | 1 | 0.5 | 0.5 | 0.5 | 0.5 | 1 | 0.5 | 0.5 | 6 |
| Fluid resuscitation in the setting of GI loss: choice of fluid, volume, rate, when to stop | 0.5 | 0.5 | 1 | 0.5 | 0.5 | 0.5 | 0.5 | 1 | 0.5 | 0.5 | 6 |
| IV fluid challenge: indications, choice of fluid, volume, rate, when to stop | 0.5 | 0.5 | 1 | 0.5 | 0.5 | 0.5 | 0.5 | 1 | 0 | 0.5 | 5.5 |
| Fluid restriction: Volume to restrict, timing, utilization of supplementation, indications, when to stop. | 0.5 | 0.5 | 1 | 0.5 | 0.5 | 0.5 | 0.5 | 1 | 0 | 0 | 5 |
| Crystalloids vs Blood products | 0 | 0 | 0 | 0 | 0 | 0.5 | 0 | 0 | 0 | 0 | 0.5 |
| TOTAL | 3 | 2.5 | 6 | 2.5 | 3 | 3.5 | 3 | 6 | 1.5 | 2.5 | 33.5 |

Legend: AB: AMBOSS; ETG: Electronic Therapeutic Guidelines; FA: First Aid; MTB: Master the Boards;

NICE: National Institute for Health and Care Excellence; OME: OnlineMedEd; OX: Oxford Handbook of Clinical Medicine;

SU: Step-Up to Step 2CK; TN: Toronto Notes

There were three possible scorings for each topic: 0 point (none covered), 0.5 points (some covered), 1 point (all covered.
